# Art in Medicine

**Published:** 1905-12-02

**Authors:** 


### Art in Medicine.

Under the suggestive title of "The Decline of Art in Medicine" Sir Dyce Duckworth has published in pamphlet form a very earnest and thoughtful address, which he recently delivered at the opening of the session of the Abernethian Society of St. Bartholomew's, and in which he struck a note well deserving of the best consideration of the profession. The general contention of the address is that while the sciences on which medicine is based have made astounding progress in the last half century, the art of medicine itself has materially declined and fallen into neglect during the same period; and this declension is attributed to the alleged circumstance that, under our present system of education, an undue amount of time is occupied by the study of scientific subjects during what should be a purely medical and surgical curriculum. Sir Dyce declares that, if pursued too far, or in wrong directions, these studies have little bearing on the student's life work; and adds that, if it be urged that they constitute important elements for his mental training, they should be unnecessary if he has already had a good general education. In support of these views he quotes the "wise words" of Dr. Spender, of Bath, who declares that the chemist, the biologist, and the physical science man must relax their hard grips on the poor medical student, and adds that no clinical and therapeutical education can be too long or too thorough, provided that literary culture and scientific habits of thought are in the background. The proviso thus made will be felt by many people to be fatal to the conclusion which it is apparently intended to establish; inasmuch as the main complaint of the "chemist, the biologist, and the physical science man" is that literary culture and scientific habits of thought, so far from being commonly found in combination, are more frequently antagonistic to each other, at least to the extent that the literary culture has left no time in which the scientific habits of thought could be acquired. It is the average man of "literary culture," who has received a good "general education," who ordinarily exhibits the most absolute inability to grasp the conditions of a scientific problem, and who best illustrates Faraday's description of the multitudes who are ready to draw conclusions when they have little or no power of judgment in the cases. Men of "literary culture," but destitute of scientific training, furnish the most conspicuous examples of incapacity to observe accurately and of inability to reason correctly, which we witness every day in the world around us. They are the patrons of quack medicines and of "anti" societies. They are apt to dogmatise serenely concerning matters of the very elements of which they are profoundly ignorant. The statesman of whom it was said that he would be equally confident in his ability to cut a man for the stone, to deliver a woman in labour, or to command the Channel Fleet, was a man of undoubted literary culture; but "scientific habits of thought" were, in his case, not so much in the background as positively non-existent. We are in much sympathy with a great deal that Sir Dyce Duckworth advances, but we cannot depart from the conclusion that a sound training in physical science is essential to the power of observing and of reasoning upon phenomena, and that it constitutes the best foundation upon which a future clinical training can be built. We agree with him that, "with the support of increasing clinical experience, attained by careful observation and an unbiassed judgment, we may stand firm amidst the strange and varied phases of opinion which prove seductive, each in its turn, to those who have failed to acquire proficiency in our art." But how are we to teach the art of careful observation? and how is the judgment to be kept "unbiassed," if it be not by the aid of those mental habits which the pursuit of physical science is eminently calculated to foster and to maintain?

